# Tunable Polyglycerol-Based Redox-Responsive Nanogels for Efficient Cytochrome C Delivery

**DOI:** 10.3390/pharmaceutics13081276

**Published:** 2021-08-17

**Authors:** Sebastian Schötz, Felix Reisbeck, Ann-Cathrin Schmitt, Mathias Dimde, Elisa Quaas, Katharina Achazi, Rainer Haag

**Affiliations:** Institute of Chemistry and Biochemistry, Freie Universität Berlin, Takustr. 3, 14195 Berlin, Germany; bastischoetz@zedat.fu-berlin.de (S.S.); freisbeck@zedat.fu-berlin.de (F.R.); schmia92@zedat.fu-berlin.de (A.-C.S.); matze111@zedat.fu-berlin.de (M.D.); equaas@zedat.fu-berlin.de (E.Q.); kachazi@zedat.fu-berlin.de (K.A.)

**Keywords:** nanogels, protein delivery, iEDDA

## Abstract

The sensitivity of therapeutic proteins is a challenge for their use in biomedical applications, as they are prone to degradation and opsonization, thus limiting their potential. This demands for the development of drug delivery systems shielding proteins and releasing them at the site of action. Here, we describe the synthesis of novel polyglycerol-based redox-responsive nanogels and report on their potential as nanocarrier systems for the delivery of cytochrome C (CC). This system is based on an encapsulation protocol of the therapeutic protein into the polymer network. NGs were formed via inverse nanoprecipitation using inverse electron-demand Diels–Alder cyclizations (iEDDA) between methyl tetrazines and norbornenes. Coprecipitation of CC led to high encapsulation efficiencies. Applying physiological reductive conditions of l-glutathione (GSH) led to degradation of the nanogel network, releasing 80% of the loaded CC within 48 h while maintaining protein functionality. Cytotoxicity measurements revealed high potency of CC-loaded NGs for various cancer cell lines with low IC_50_ values (up to 30 μg·mL^−1^), whereas free polymer was well tolerated up to a concentration of 1.50 mg·mL^−1^. Confocal laser scanning microscopy (CLSM) was used to monitor internalization of free and CC-loaded NGs and demonstrate the protein cargo’s release into the cytosol.

## 1. Introduction

Therapeutic proteins have attracted growing interest due to their increased specificity and versatility of use in biological processes. Compared to small organic molecules, proteins do not have solubility problems for medical applications, but they suffer from proteolytic aggregation at surfaces, as well as physical unfolding under physiological conditions [1,2]. This results in very poor oral bioavailability and pharmacokinetic properties, making intravenous injection inevitable [3]. Once in the bloodstream, proteins often exhibit a short circulation time due to enzymatic degradation by proteases, immune recognition, and rapid renal clearance. Small proteins with molecular weights below the renal threshold, considered to be 50–70 kDa, show particularly fast renal clearance, resulting in elimination from the bloodstream [4,5]. To overcome this barrier, a common strategy is to covalently attach a polyethylene glycol (PEG) chain to the protein. This increases the molecular weight, leading to longer circulation times, and shields the protein from external stimuli [6]. Based on this strategy, several PEGylated proteins have been developed and certified by the FDA as therapeutics for diseases such as hemophilia A, phenylketonuria, and acute lymphoblastic leukemia [7,8,9]. However, PEG was found to be recognized by the human immune system, leading to immunogenic reactions such as ocular irritation, respiratory compromise, or anaphylaxis [10,11]. In order to overcome these hurdles, research has focused on developing alternative polymers to replace the gold-standard PEG [12]. Another promising avenue is the development of nanosized drug delivery systems (DDSs) such as nanogels (NGs) that can physically mask and encapsulate therapeutic proteins. This approach requires no chemical modification of the protein.

NGs are highly water-swollen, crosslinked, three-dimensional networks of hydrophilic or amphiphilic polymers in the size range of 10–1000 nm [13,14]. Due to their ability to swell in aqueous media, hydrophilic polymers such as PEG, polyvinyl alcohol (PVA), dendritic polyglycerol (dPG), chitosan, and alginate have been reported as polymeric precursors [15,16,17,18,19]. In many cases they show low and nonspecific interactions with blood proteins, making them promising candidates for tissue engineering, biomedical applications, drug delivery, and biotechnology [20,21,22,23]. NGs can be produced using a variety of methods. Commonly, mini- and microemulsion polymerizations are used to obtain droplets that act as nanoreactors [24,25]. However, to guarantee the formation and stability of these droplets, surfactants and ultrasonication are required. Since both can lead to denaturation of proteins during in situ encapsulation, this approach is not suitable for the synthesis of a DDS with protein cargo. As an alternative, our group recently reported on inverse nanoprecipitation as a procedure to synthesize dPG-based NGs under mild conditions in the absence of any additives [26]. This method relies on using a nonsolvent that diffuses into the dissolved macromonomer solution, leading to the formation of nanoaggregates [27]. In these nanoaggregates, the macromonomers are crosslinked to form a nanosized polymer network with an overall narrow size distribution. Based on this procedure, various requirements result for a suitable crosslinking strategy, which can be summarized under the term biorthogonality [28]. The reaction itself must be carried out quickly under mild conditions. No byproducts should be formed that could induce denaturation, and the reactive components should not interact with other groups.

Based on their reaction kinetics, click-type reactions such as the thiol-Michael addition [29,30], strain-promoted azide alkyne addition (SPAAC) [31], and inverse electron-demand Diels–Alder (iEDDA) cyclization [32] can be used for network formation during inverse nanoprecipitation. Although the thiol-Michael addition is fast and scalable, it is not a suitable crosslinking strategy for thiol-containing proteins due to potential side reactions. SPAAC uses precursors that require a long, expensive, and low-yielding synthetic route. We earlier reported the formation of dPG-based NGs using iEDDA reactions between easily accessible methyl tetrazines and norbornene derivatives. With this method, we were able to obtain NGs with a tunable size and narrow PDI in the absence of any additives, offering great potential for protein encapsulating NGs for DDSs [33].

In this work, we present the synthesis of new dPG-based redox-responsive NGs for the intracellular delivery of cytochrome C (CC), a protein that is apoptotic when released into the cytosol. We obtained NGs via iEDDA-mediated inverse nanoprecipitation. Redox responsiveness was introduced by using a cystamine-based linker functionalized with norbornene on both sites. Release of CC under reductive conditions showed excellent protein activity. As a proof of concept, we investigated the cell viability of the loaded compared to the free NGs and their internalization for different cell lines. Unloaded NGs showed no cytotoxic effects at concentrations of up to 1.50 mg·mL^−1^, whereas the CC-loaded NGs exhibited high potency against all three cell lines, with low IC_50_ values. Overall, these NGs show excellent biocompatibility, biodegradability, high tunability with a narrow size distribution, and good stability under acidic, neutral, and basic conditions. This makes our system a promising candidate for a delivery platform of therapeutic proteins in possible future biomedical applications.

## 2. Materials and Methods

### 2.1. Chemicals and Reagents

Acetamidine hydrochloride, zinc triflate, 4-cyanobenzoic acid, and hydrazine were purchased from Sigma-Aldrich (Merck KGaA, Darmstadt, Germany). 1-Ethyl-3-(3-dimethylaminopropyl)carbodiimid-hydrochlorid(EDC·HCl) was purchased from Carl Roth (Carl Roth GmbH + Co. KG, Karlsruhe, Germany). 1-[bis(Dimethylamin)methylen]-1*H*-1,2,3-triazol[4,5-*b*]pyridinium-3-oxid-hexafluoro-phosphate (HATU) was purchased from TCI (TCI Deutschland GmbH, Eschborn, Germany). 4-(Dimethylamino)pyridin (DMAP), bicyclo[2.2.1]hept-5-en-2-ylmethanol, 4-nitrophenyl chloroformate, and cystamine hydrochloride were purchased from Sigma-Aldrich. Dichloromethane (DCM) and ethyl acetate were purchased in high-performance liquid chromatography (HPLC) grade from Sigma-Aldrich and used without any further purification. l-glutathione (GSH) and fluorescein isothiocyanate (FITC) were purchased from TCI. Cyclohexane was obtained in technical pure grade from Sigma-Aldrich and used without any further purification. Dry DCM, *N*,*N*-dimethylformamide (DMF), pyridine, and methanol (MeOH) were purchased from Sigma-Aldrich. Water was used from a Milli-Q station from Millipore (Merck KGaA, Darmstadt, Germany). All reactions were performed under an argon atmosphere using standard Schlenck techniques and oil pump vacuum.

### 2.2. Instrumentation and Methods

#### 2.2.1. Characterization Methods

Qualitative thin-layer chromatography (TLC) was carried out on silica-coated aluminum plates (silica gel 60 F254) that were obtained from Macherey Nagel (MACHEREY-NAGEL GmbH & Co. KG, Düren, Germany). Analysis of the TLC was done by fluorescence quenching under UV-light (254 nm) or by a stain of potassium-permanganate- (100 mL deionized water, 200 mg potassium permanganate or anisaldehyde-based (450 mL ethanol (EtOH), 25.0 mL anisaldehyde, 25.0 mL conc. sulfuric acid, 8.00 mL acetic acid) purchased from Sigma-Aldrich and heat as developing agents.

Medium-pressure liquid chromatography (MPLC) was performed with a TELEDYNE ISCO Combi-Flash FR 200 from TELEDYNE (Teledyne Technologies Incorporated, Lincoln, USA). Prepacked SiO_2_ columns and cartridges were obtained from TELEDYNE. As eluants, cyclohexane (99.5% quality) and ethyl acetate (HPLC grade) were used. All dialyses were performed in regenerative cellulose (RC) dialysis tubes with a molecular weight cutoff (MWCO) of 1 kDa and 100 kDa purchased from Sigma-Aldrich and Fisher Scientific (Fisher Scientific GmbH, Schwerte, Germany).

^1^H-NMR spectra were recorded at 300 K on a Joel ECX 400 MHz (JEOL Ltd., Tokyo, Japan) or a Bruker Avance III 700 MHz (Bruker Corporation, Billerica, MA, USA) in the reported deuterated solvents. Chemical shifts δ are indicated in parts per million (ppm) with reference to the residual solvent peaks. To designate multiplicities, the following abbreviations were used: s = singlet. In case that no multiplicity could be identified, they were stated as m = multiplet and their chemical shift range of signal was given. ^13^C-NMR spectra were recorded at 300 K on a Bruker Avance III (176 MHz). Chemical shifts δ are indicated in parts per million (ppm) with reference to the residual solvent peaks. The spectra were decoupled from proton broadband.

Fourier transform infrared spectroscopy (FTIR) was performed on a JASCO FT/IR spectrometer (JASCO Deutschland GmbH, Pfungstadt, Germany) via attenuated total reflectance (ATR). Wave numbers $\tilde{\nu }\;$ are given in cm^−1^.

High-resolution mass spectra (HRMS) were obtained from an AGILENT 6210 ESI-TOF spectrometer (Agilent Technologies Germany GmbH & Co. KG, Frankfurt am Main, Germany).

Dynamic light scattering (DLS) and zeta potential were measured on a Malvern zeta-sizer nano ZS ZEN 3600 ((Malvern Instruments Ltd., Worcestershire, UK)) using an He–Ne laser (λ = 532 nm) at 173° backscatter and automated attenuation at, unless stated otherwise, 25 °C. All sample measurements were performed in triplicate, yielding a mean size value plus standard deviation. All measurements were performed in triplicate.

Gel permeation chromatography (GPC) was measured on an AGILENT 1100 at 5 mg·mL^−1^ sample concentration using a pullulan standard, 0.1 M NaNO_3_ solution, as an eluant, and a PSS Suprema column (PSS Polymer Standards GmbH, Mainz, Germany) 10 μm with a flow rate of 1 mL·min^−1^. The signals were detected with an RI detector from PSS Polymer Standards.

UV/Vis measurements were conducted on an AGILENT Cary 8454 UV–visible spectrophotometer, using half-micro quartz cuvettes.

For the preparation of cryo-sample droplets, 4 µL of the sample solution was placed on hydrophilized holey carbon filmed grids (Quantifoil R1/2 from Quantifoil Micro Tools GmbH, Großlöbichau, Germany) at room temperature. The excess fluid was blotted off using filter paper to generate an ultrathin layer of the solution (typical thickness around 100 nm) spanning the holes in the carbon film. The grids were vitrified in liquid ethane using an automated vitrification robot (FEI Vitrobot Mark III from Fisher Scientific) and stored in liquid nitrogen prior to the measurement.

Cryogenic transmission electron microscopy (cryo-TEM) was carried out using a Talos Arctica transmission electron microscope (Thermo Fisher Scientific Inc., Waltham, USA). The vitrified grids were stabilized by a copper autogrid and fixed with a spring clamp under liquid nitrogen. These autogrids were transferred under liquid nitrogen into the transmission electron microscope using the microscope’s autoloader transfer routine.

Micrographs were recorded using the microscopes low-dose protocol at a primary magnification of 28,000× and an acceleration voltage of 200 kV. Images were recorded by a Falcon 3CE direct electron detector (48 aligned frames) at full size (4k) from Thermo Fisher. The defocus was chosen to be 5 µm in all cases to create sufficient phase contrast.

#### 2.2.2. Synthesis of Starting Materials

The reported spectra can be found in the Supplementary Materials.

##### Synthesis of dPG (**1**) and dPG-NH_2_ (**2**)

Both compounds were synthesized according to the literature [12,34].

##### 4-(6-Methyl-1,2,4,5-tetrazin-3-yl)benzoic Acid (**4**)

The synthesis of 4-(6-methyl-1,2,4,5-tetrazin-3-yl)benzoic acid (**4**) was performed according to a reported procedure [33]. In brief, 4-cyanobenzoic acid (**3**) (1.50 g, 10.0 mmol), acetamidine hydrochloride (4.82 g, 41.0 mmol), and Zn(OTf)_2_ (1.00 g, 3.00 mmol) were ground in a mortar, added to a 100 mL Schlenk flask under argon atmosphere, cooled to 0 °C, and dissolved in anhydrous hydrazine (12.0 mL, 377 mmol). The ice bath was removed, and the solution was allowed to warm to room temperature. After 72 h of stirring, NaNO_2_ (10.0 g), dissolved in deionized water, was added. After cooling to 0 °C, the pH was adjusted to 2 using conc. hydrochloric acid. The resulting precipitate was filtered and washed with deionized water and MeOH to obtain the product (**4**) as a pink solid (1.10 g, 50%).

^1^H-NMR (400 MHz, C_3_D_7_NO): δ = 8.68–8.66 (m, 2H), 8.31–8.29 (m, 2H), 3.09 (s, 3H) ppm.

##### dPG-metTet **M_1_**

4-(6-Methyl-1,2,4,5-tetrazin-3-yl) benzoic acid (**4**) (0.89 g, 4.05 mmol) was dissolved in dry DMF (50 mL). DMAP (0.65 g, 5.41 mmol), EDC·HCl (1.04 g, 5.41 mmol) and HATU (2.06 g, 5.41 mmol) were added, and the solution was stirred at room temperature for 1 h. Meanwhile, dPG-NH_2_ (**2**) (4.00 g, 6 kDa, dF = 10%) was dissolved in dry DMF (50 mL), while DMAP (0.65 g, 5.41 mmol) and dry pyridine (0.43 g, 5.41 mmol, 0.44 mL) were added. After 1 h, the solution of (**4**) was added dropwise via syringe to the solution of (**2**) and stirred for 24 h at room temperature. The crude product was dialyzed against MeOH for 5 days (MWCO = 1 kDa) to obtain dPG-metTet (**M_1_**) in methanolic solution (dF = 6.5%, 85%).

^1^H-NMR (700 MHz, MeOD): δ = 8.69–8.53 (m, 2 H), 8.12–7.98 (m, 2 H, H–2), 4.05–3.48 (dPG–backbone), 3.10 (s, 3 H) ppm. ^13^C-NMR (176 MHz, MeOD): δ = 169.4, 169.2, 164.8, 139.1, 136.3, 129.4, 128.9, 81.7, 81.4, 80.2, 79.8, 74.0, 73.0, 72.5, 72.2, 71.0, 70.7, 70.3, 64.5, 64.4, 62.8 ppm. IR (ATR): $\tilde{\nu }$ = 3348, 2871, 1644, 1548, 1456, 1404, 1364, 1327, 1305, 1258, 1070, 931 cm^−1^.

##### Bicyclo[2.2.1]hept-5-en-2-ylmethyl (4-nitrophenyl) Carbonate (**6**)

The carbonate (**6**) was synthesized according to a literature protocol [33]. Bicyclo[2.2.1]hept-5-en-2-ylmethanol (**5**) (3.00 g, 24.2 mmol) was dissolved in dry DCM (80 mL). 4-Nitrophenyl chloroformate (7.30 g, 36.24 mmol) and pyridine (2.49 g, 31.5 mmol, 2.53 mL) were added. The solution was stirred at room temperature for 12 h. The reaction was quenched with saturated NH_4_Cl_aq_ (200 mL), the phases were separated, and the aqueous phase was extracted with DCM (3 × 100 mL). The combined organic layers were dried over Na_2_SO_4_, and the solvent was removed under reduced pressure. The crude product was purified via MPLC (cyclohexane/ethyl acetate 10:1) to obtain the product (**6**) as a white solid (6.09 g, 21.1 mmol, 90%).

^1^H-NMR (400 MHz, MeOD): δ = 8.32–8.29 (m, 2H), 7.49–7.45 (m, 2H), 6.29–5.98 (m, 2H), 4.38–3.85 (m, 2H), 2.93–2.77 (m, 2H), 2.56–2.50 (m, 1H), 1.94–0.60 (m, 4H)) ppm.

##### Norb-Cys-Norb **M_2_**

Cystamine hydrochloride (0.65 g, 2.90 mmol) was dissolved in a mixture of dry methanol and dry DMF (ratio 1:2, 20 mL). Triethylamine (1.50 g, 14.5 mmol, 2.02 mL) and norbornene carbonate (**6**) (7.26 mmol, 2.10 g) were added, and the solution was stirred at room temperature for 8 h. The reaction was quenched with aqueous saturated NaHCO_3_-solution (20 mL). The phases were separated, and the aqueous phase was extracted with EtOAc (3 × 50 mL). The combined organic layers were dried with Na_2_SO_4_, the solvent was evaporated under reduced pressure, and the crude product was purified via MPLC (cyclohexane/EtOAc 10:1 to 4:1) to obtain the carbamate Norb-Cys-Norb (**M_2_**) (1.18 g, 2.61 mmol, 36%) as a yellow solid.

^1^H-NMR (700 MHz, MeOD): δ = 6.18−5.96 (m, 4H), 4.14−3.60 (m, 4H), 3.43−3.40 (m, 4H), 2.89−2.72 (m, 8H), 2.40−1.85 (m, 1 H), 1.72−0.55 (m, 8H) ppm. ^13^C-NMR (176 MHz, MeOD): δ = 159.2, 138.5, 137.9, 133.2, 70.0, 69.3, 50.3, 45.8, 45.1, 44.8, 43.5, 42.8, 41.0, 39.7, 39.4, 39.2, 30.3, 29.7, 28.0 ppm. HRMS (ESI, pos. mode): calc. for C_22_H_33_N_2_O_4_S_2_^+^ [M + H]^+^: 453.1876, found: 453.1834; calc. for C_22_H_33_N_2_O_4_S_2_Na^+^ [M + Na]^+^: 475.1695, found: 475.1679; calc. for C_22_H_33_N_2_O_4_S_2_K^+^ [M + K]^+^: 491.1435, found: 491.1412.

#### 2.2.3. Nanogel Synthesis

NGs were synthesized via inverse nanoprecipitation. The ratio of the precursor **M_1_** (dPG-metTet) to **M_2_** (Norb-Cys-Norb) was set to 1:1.5. The standard batch size was set to 5 mg·mL^−1^. Acetone was used as nonsolvent. As an example, a general procedure for the preparation a NG is described below.

**M_1_** was stored in a stock solution (150 mg·mL^−1^) in water and **M_2_** was stored as a stock solution (50 mg·mL^−1^) in acetone. An aliquot was taken and separately diluted with water to a final volume of 1 mL. For this, 30 μL of **M_1_** was diluted with 470 μL of water, and 10 μL of **M_2_** was diluted with 490 μL of acetone. Both solutions were cooled in an ice bath to 4 °C. **M_2_** was added quickly to the solution of **M_1_** via a syringe, briefly vortexed for 5 s, and injected quickly via a syringe into a 30 mL glass vial, containing a solution of 20 mL of acetone under vigorous stirring (1100 rpm). The suspension was stirred for another s and then kept still. After 30 min, 10 mL of phosphate buffer solution (10 mM, pH = 7.4) and 20 μL of 2-(vinyloxy)-ethan-1-ol were added under stirring (1100 rpm.). The solution was transferred into a 50 mL round-bottom flask, acetone was evaporated, and the NGs were obtained as stable dispersions in an aqueous medium with an average yield of 95%. Characterization was performed by means of DLS, NMR, and cryo-TEM measurements.

#### 2.2.4. Fluorescein Isothiocyanate (FITC) Labeling of M_1_

**M1** (0.12 g, 1.62 mmol) was dissolved in dry DMF (10 mL). FITC (0.010 g, 0.24 mmol) was added, and the solution was stirred for 48 h at 60 °C. The solution was cooled to room temperature and dialyzed against deionized water for 48 h to obtain the FITC–dPG-metTet (0.10 g, 84%).

UV/Vis (H_2_O): λ_max_ (ϵ): 500 nm (1 mg·mL^−1^).

#### 2.2.5. Coprecipitation of CC

CC was stored in a 5 mg·mL^−1^ stock solution of water. Then, 125 μL up to 500 μL of protein solution was added to the aqueous solution of **M_1_** depending on the targeted protein loading content. Inverse nanoprecipitation was performed as described in the NG formation section. The resulting solution was dialyzed (MWCO = 100 kDa) against PB (10 mM) for 24 h. All encapsulation experiments were performed in triplicate. Protein loading content and protein loading efficiency were determined via UV/Vis, using the absorbance of CC at 410 nm.

#### 2.2.6. Degradation of Unloaded NGs

To a solution of the NGs (2 mg·mL^−1^, 1 mL in PB (10 mM, pH 7.4)), GSH (10 mM) was added and the pH was adjusted to 7.4. The resulting solution was incubated for 2 days at 37 °C. As a control, a NG sample without GSH was prepared accordingly. DLS measurements were performed after 0, 1, and 2 days.

#### 2.2.7. Release of CC

The release of CC from the NGs was performed at 37 °C in PB (10 mM, pH = 7.4) or PB (10 mM, pH = 7.4) with 10 mM GSH in triplicates.

First, 1 mL of dPG-NGs loaded with CC (CC@NGs) (1.25 mg·mL^−1^) were placed in a dialysis tube (MWCO = 100 kDa) with 20 mL of release medium. At each time stamp, 5 mL of medium was withdrawn, and 5 mL of fresh release medium was refilled. The released amount of CC was quantified via UV/Vis spectroscopy using the protein absorbance at 410 nm and calculated on the basis of a calibration curve of the protein.

#### 2.2.8. Determining Enzymatic Activity of CC

The activity of CC was determined via ABTS assays, as described in the literature [35].

For this, CC was taken from the release medium described in Section 2.2.8, diluted to 1 mL at a concentration of 4 μg·mL^−1^, and transferred to a quartz cuvette. Next, 10 μL of H_2_O_2_ (0.045 M) and 100 μL of 2,2’-azinobis(3-ethylbenzthiazoline-6-sulfonic acid) (ABTS) with a concentration of 1.00 mg·mL^−1^ were added. Then, the absorbance at 405 nm of the oxidized product was monitored via UV/Vis spectroscopy every 30 s for 180 s. As a control, native CC (4 μg·mL^−1^) and native CC (4 μg·mL^−1^) incubated with GSH (10 mM) were used and treated in the same manner.

#### 2.2.9. Cell Viability Studies

The effect of the dPG-NGs (NG) and the NG@CC, as well as free CC, on three cancer cell lines, A549, Hela, and MCF7, was determined using the cell viability assay Cell Counting Kit 8 (CCK-8) from Sigma-Aldrich Chemie GmbH (Taufkirchen, Germany) according to the manufacturer’s instructions. A549 (DSMZ no.: ACC 107), Hela (DSMZ no.: ACC 57), and MCF7 (DSMZ no.: ACC 115) cells were obtained from Leibniz-Institute DSMZ (Deutsche Sammlung von Mikroorganismen und Zellkulturen GmbH, Braunschweig, Germany) and cultured in Dulbecco’s modified Eagle’s medium (DMEM) supplemented with 10% (*v/v*) FBS, 100 U·mL^−1^ penicillin, and 100 µg·mL^−1^ streptomycin (all from Gibco BRL, Eggenstein, Germany). Cells were regularly subcultured at least twice a week when they reached 70% to 90% confluency. For the cytotoxicity assay, 90 µL of a cell suspension in DMEM containing 5 × 10^4^ cells per mL was seeded in each inner well of a 96-well plate and incubated overnight at 37 °C and 5% CO_2_. In the outer wells, 90 µL DMEM without cells were added. On the next day, serial dilutions of all the samples were prepared, and 10 µL of each was added to the cells in triplicate, in addition to one outer well for background correction. SDS (1%) and nontreated cells served as controls. After another 72 h at 37 °C and 5% CO_2_, the CCK-8 solution was added (10 µL/well), and absorbance at a measurement wavelength of 450 nm and a reference wavelength of 650 nm was measured after approximately 3 h incubation using a Tecan plate reader (infinite pro200, TECAN-reader Tecan Group Ltd., Männedorf, Switzerland). Measurements were performed in triplicate and repeated three times. The cell viability was calculated by setting the nontreated control to 100% after subtracting the background using the Excel software. All cell experiments were conducted according to German genetic engineering laws and German biosafety guidelines in the laboratory (safety level 1).

The half-maximal inhibitory concentration (IC_50_) was calculated with GraphPad Prism 6.01 (Graph Pad Software, San Diego, CA, USA) using the log (inhibitor) vs. normalized response variable slope equation.

#### 2.2.10. Cell Uptake Assay

The uptake of FITC-labeled dPG-NGs with and without CC in A549, Hela, and MCF7 cells was analyzed using confocal laser scanning microscopy (CLSM). The cells were propagated as described above. For CLSM, HeLa cells were seeded in eight-well ibidi slides (ibidi treat) in 270 µL of DMEM. After cell attachment for 4 h to 24 h, 30 µL of a post-seeding solution containing compound was added, and the cells were further incubated overnight. Before imaging, the cells were stained with Hoechst 33342 (1 µg·mL^−1^), washed twice with PBS, and covered with fresh cell culture medium (DMEM). Confocal images were taken with an inverted confocal laser scanning microscope Leica DMI6000CSB SP8 (Leica, Wetzlar, Germany) with a 63×/1.4 HC PL APO CS2 oil immersion objective using the manufacturer-given LAS X software in sequential mode with the following channel settings: transmission Ch (gray intensity values), excitation laser line 405 nm, detection of transmitted light (photomultiplier); Ch1 (Hoechst 33342): excitation laser line 405 nm, detection range 410 nm–484 nm (hybrid detector); Ch2 (FITC): excitation laser line 488 nm, detection range 493 nm–712 nm (hybrid detector).

## 3. Results and Discussion

### 3.1. Synthesis of Precursors: dPG metTet and Norb-Cys-Norb

**Scheme 1:**
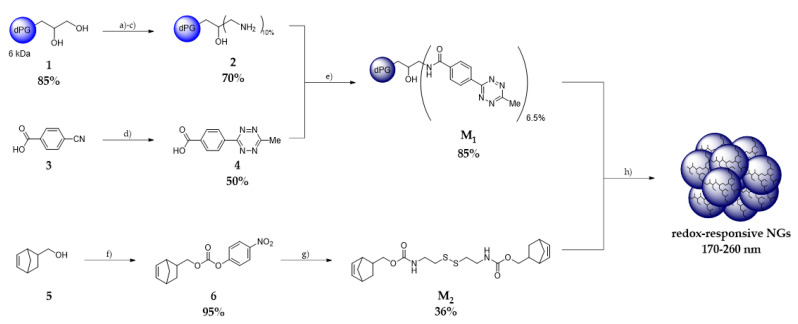
Overview of synthetic strategy to obtain redox-responsive dPG-based NGs. (**a**) MsCl, Et_3_N, DMF, room temperature, overnight; (**b**) NaN_3_, 60 °C, 3 days; (**c**) PPh_3_, water/THF, room temperature, 3 days; (**d**) acetamidine hydrochloride; Zn(OTf)_2_, N_2_H_4_, 0 °C to room temperature, 2 days, then NaNO_2_, H_2_O, HCl, 2 h; (**e**) EDC·HCl, HATU, DMAP, pyridine, DMF, room temperature, overnight; (**f**) 4-nitrophenyl chloroformate, pyridine, DCM, room temperature, 12 h; (**g**) cystamine hydrochloride, Et_3_N, DCM/MeOH, room temperature, overnight; (**h**) inverse nanoprecipitation.

Starting with dPG (**1**) [36], dPG–NH_2_ (**2**) was obtained in three steps with an overall yield of 70%, following a literature protocol via mesylation, azidation, and finally reduction under Staudinger conditions.[37] dPG–metTet (**M_1_**) was obtained via amide coupling of metTet–COOH (**4**) and dPG–NH_2_ (**2**). For the synthesis of Norb–Cys–Norb (**M_2_**), we converted bicyclo[2.2.1]hept-5-en-2-ylmethanol (**5**) into its reactive carbonate derivative (**6**) by using 4-nitrophenyl chloroformate. This allowed carbamate coupling of norbornene to the amines of cystamine under mild conditions, yielding Norb–Cys–Norb (**M_2_**) with an overall yield of 35%. Both precursors were further used for NG formation via inverse nanoprecipitation (Scheme 1).

### 3.2. Formation and Characterization of NGs

Inverse nanoprecipitation was performed by mixing **M_1_**, dissolved in an aqueous solution and **M_2_** dissolved in acetone. The resulting mixture was injected into acetone, and then water was added to obtain NGs as a stable dispersion in water (Scheme 1). Their crosslinked structure was proven by ^1^H-NMR spectroscopy, as depicted in Figure S1. The tunability of the hydrodynamic diameter was investigated by increasing the amount of linker (**M_2_**) from 0.25 to 2.00 equivalents. The obtained NGs were analyzed by DLS. This procedure yielded NGs in a range of 171 nm to 257 nm with a narrow dispersity (PDI > 0.11) and a slightly positive surface charge that derived from the remaining amine groups of dPG–metTet (Table 1).

**Figure 1:**
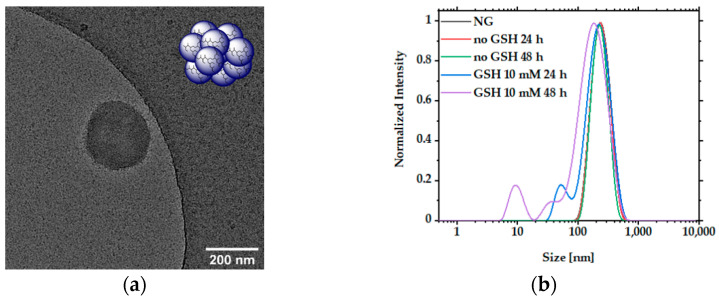
Analysis of obtained nanogels (NGs). (**a**) Size of NGs as measured by cryo-TEM. The full cryo-TEM is depicted in Figure S2. (**b**) Change in size distribution of NGs after incubation at 37 °C in 10 mM phosphate buffer (PB) with and without addition of 10 mM l-glutathione (GSH) at pH 7.4.

Cryo-TEM revealed the spherical appearance of the free NGs, with a diameter of 240 nm (Figure 1a). The NGs showed excellent stability and only minimal changes in the size distribution curve after incubation at 37 °C in PB solution. Degradation products with sizes < 100 nm and a shift of the main peak to smaller size values could be observed after 24 h and 48 h of incubation with 10 mM GSH, indicating cleavage of the disulfide bonds (Figure 1b) and supporting the stated redox-responsiveness of the NGs. As another control, experiments were repeated with GSH with a dPG–metTet and dPG–Norb-based system that was published previously [33], which does not contain any disulfide bonds. Here, no change in the size distribution could be observed over a period of 48 h (see Figure S3).

**Figure 2:**
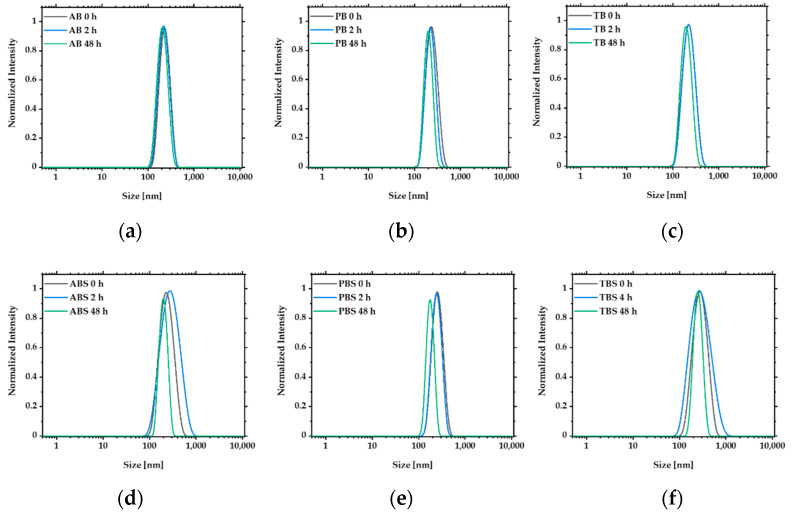
Stability studies of NGs in different buffer solutions measured by DLS. NGs were incubated at 37 °C in three different buffer solutions (10 mM) with and without saline (140 mM) for 48 h. (**a**) pH 4.5 acetate buffer (AB); (**b**) pH 7.4 phosphate buffer (PB); (**c**) pH 8.1 Tris buffer (TB); (**d**) pH 4.5 acetate-buffered saline (ABS); (**e**) pH 7.4 phosphate-buffered saline (PBS); (**f**) pH 8.1 tris-buffered saline (TBS).

The stability of the obtained NGs was studied by transferring the particles into different buffer solutions with pH values in a range of 4.5 to 8.1. Incubation in the absence of saline revealed that the size distribution remained constant over a period of 48 h, with only minimal changes in their hydrodynamic diameter. For the saline-containing buffers, interactions between the hydrogel network and the increased salt content were observed. This dynamic resulted in a broader size distribution over time for the acidic and basic buffer (Figure 2c,d) and a hydrodynamic shift to smaller values for the neutral condition (Figure 2e). No degradation was detected for any of the tested conditions. The evident stability of these NGs ensures safe and stable encapsulation of biomolecules under physiological conditions.

### 3.3. Encapsulation and Release of CC

**Figure 3:**
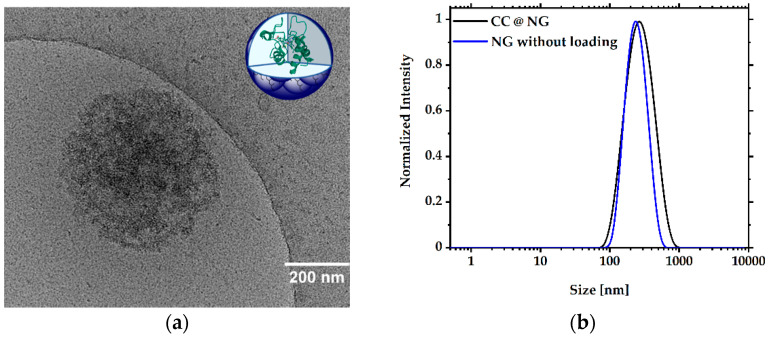
Characterization of cytochrome C-loaded NGs (CC@NGs). (**a**) Cryo-TEM image of CC@NGs with a scale bar of 200 nm. The full cryo-TEM is depicted in Figure S4. (**b**) Size distribution curves of CC@NGs (black) compared to free nanogel (NG) (blue) measured by DLS.

NGs were loaded with CC via coprecipitation during inverse nanoprecipitation. To determine the protein loading capacity (PLC) and protein loading efficiency (PLE), loading experiments with different protein loading contents ranging from 5–20 wt.% were performed. A maximum PLC of 8.7%, with an efficiency of 87%, was obtained using 10 wt.% CC. Increasing the amount of CC led to similar PLC values, indicating protein saturation of the system at 9 wt.% PLC. For the saturated CC@NGs, a narrow PDI of >0.2 with a neutral surface charge was obtained (Table 2). DLS measurements showed a shift in the mean size values to around 400 nm, an increase as compared to the unloaded NGs (Figure 3b). Cryo-TEM measurements of CC-loaded NGs (CC@NGs) revealed a spherical appearance with a diameter of 360 nm. Compared to the unloaded NGs (Figure 1a), we observed an increased electron density in their CC-loaded analogues. We assume that this is due to the protein cargo, proving successful encapsulation of CC (Figure 3a).

**Figure 4:**
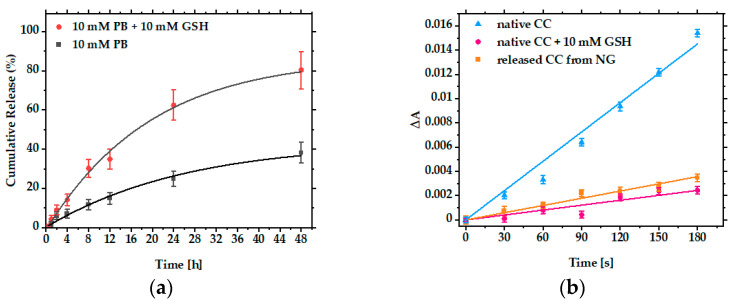
(**a**) Release of cytochrome C (CC) from loaded nanogels (NGs) with addition of l*-*glutathione (GSH) (blue) and control in buffer (black). The release was carried out at 37 °C in 10 mM phosphate buffer (PB) at pH 7.4. (**b**) Oxidative ABTS assay with native CC (blue), GSH-treated native CC (red), and CC released from NGs (orange).

In tumor cells, an increased concentration of GSH (2–10 mmol) can be found [38]. Therefore, the release of CC under reductive conditions (10 mM GSH) at 37 °C and pH 7.4 was investigated. A sample not treated with GSH was used as a control. After 48 h of incubation at pH 7.4 and 37 °C, 80% of the loaded CC was found in the release medium, compared to 35% in the control (Figure 4a). This supports the assumption that the disulfides embedded in the network structure of the NG are cleaved under physiological reductive conditions, ultimately leading to the release of the protein. ABTS assays of the released and native CC showed similar enzymatic activity in a reductive environment, indicating full functionality of the protein after coprecipitation and in vitro release (Figure 4b). Li et al. used a similar reductively cleavable NG system based on hyaluronic acid for the intracellular delivery of CC. With this system, they were able to release 90% of the loaded CC after 24 h of GSH incubation, while preserving protein functionality. Interestingly, their nontreated control released less than 20% of the protein cargo. We believe that, after NG formation, the protein is physically trapped in the network due to the mesh size of the polymeric carrier. As the NG network is not a static system and the obtained meshes are not equally sized over the whole network, leaching of the protein is possible, leading overall to a protein release without applying reductive conditions. A charged polymer network such as hyaluronic acid can decrease this process due to secondary interactions with the protein cargo. We assume that this is the reason for the increased protein release of our control (Figure 4a) compared to the results that were obtained by Li et al. [35].

### 3.4. Cell Viability Studies and Cellular Uptake

**Figure 5:**
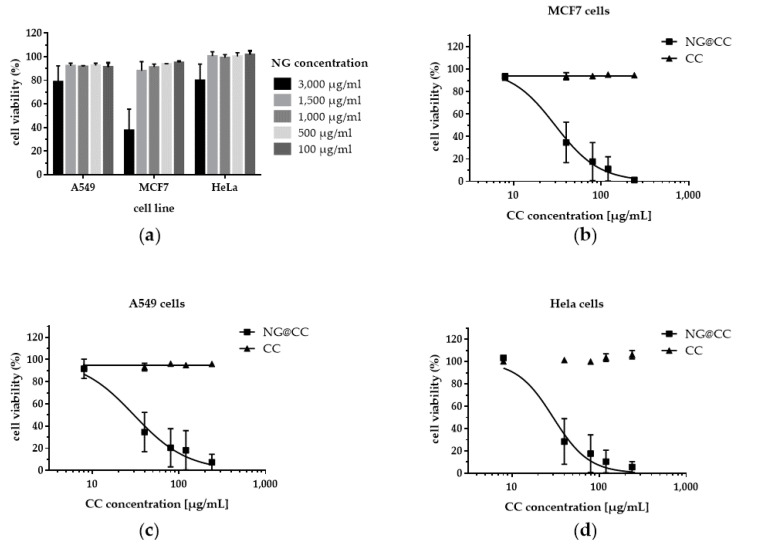
Cell viability results obtained from the cell viability assay (CCK-8) upon incubation of A549, HeLa, and MCF7 cells. (**a**) Cell viability of unloaded nanogels (NGs) tested for A549, McF7, and HeLa cells after 72 h of treatment. (**b)** Cell viability of cytochrome C-loaded nanogels (NG@CC) with a protein loading content of 9% and free cytochrome C (CC) tested on McF7 cells after 72 h of treatment. (**c**) Cell viability of cytochrome C-loaded nanogels (NG@CC) with a protein loading content of 9% and free cytochrome C (CC) tested on A549 cells after 72 h of treatment. (**d**) Cell viability of cytochrome C-loaded nanogels (NG@CC) with a protein loading content of 9% and free cytochrome C (CC) tested on HeLa cells after 72 h of treatment. The cell viability was normalized to 100% using the nontreated control. Measurements were performed in triplicates and repeated three times. Data are represented as the mean of the three independent experiments with standard deviation.

Cytotoxicity assays of HeLa, McF7, and A549 revealed a high cell viability for the free NGs up to a concentration of 1.50 mg·mL^−1^, as well as for free CC up to a concentration of 0.24 mg·mL^−1^. CC-loaded NGs in the same concentration caused a significant decrease in cell viability in all three cell lines at a loaded CC concentration of 30 μg·mL^−1^. The results indicate cellular uptake of the loaded NGs and subsequent release of CC to the cytosol, a finding reinforced by the obtained low IC_50_ from these measurements (Figure 5, Table 3). We hypothesize that this is due to the fact that the uptake of free CC is drastically reduced as compared to its NG-loaded analogue. This is in line with previous experiments using FITC-labeled CC [39], as well as with the results obtained by Li et al. with their system [35].

**Figure 6:**
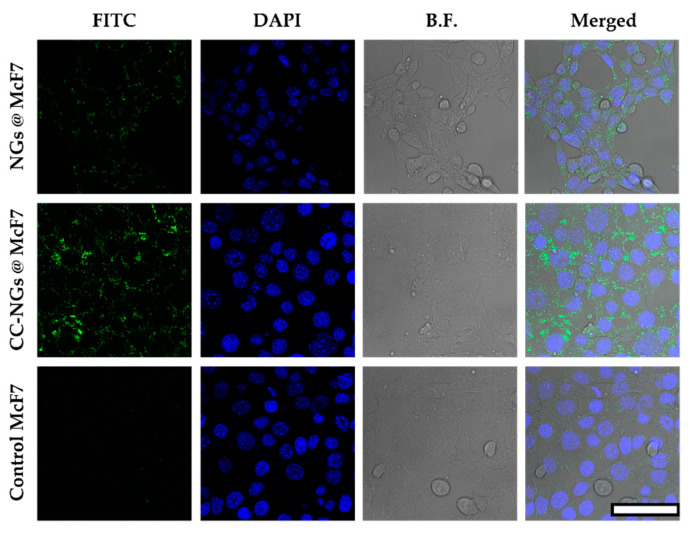
Confocal laser scanning microscopy (CLSM) of McF7 cells. Nuclei were stained with Hoechst dye (blue). Cells were incubated for 16 h at 37 °C with 0.5 mg·mL^−1^ of loaded and unloaded FITC-labeled NGs (green). Untreated cells were measured as a control (scale bar indicates 75 μm).

As cell viability studies indicated cell uptake of the NGs and a subsequent release of CC, we then further monitored the uptake of NGs by means of confocal laser scanning microscopy (CLSM). For the CLSM study, **M_1_** was labeled with FITC prior to NG formation. Overnight incubation of Hela, MCF7, and A549 cells with the FITC-labeled NGs, with and without CC cargo, revealed efficient cellular uptake of the loaded and unloaded nanoparticles, as depicted in Figure 6 (as well as Figures S5 and S6 for A549 and HeLa, respectively).

## 4. Conclusions

This research demonstrated the synthesis of dPG-based methyl tetrazines and cystamine-based norbornenes. We were able to reproducibly obtain NGs with dispersity indices of 0.11 and below. The prepared NGs retained their size under acidic, neutral, and basic conditions, indicating high stability of the hydrogel network under physiological conditions. Coprecipitation of CC revealed high encapsulation efficiencies of more than 80%, with up to 10 wt.% protein feed ratios. Applying reductive conditions led to degradation of the NGs and release of 80% of loaded CC within 48 h. ABTS assays revealed full functionality of the protein cargo after loading and in vitro release. Cell viability studies on McF7, HeLa, and A549 cell lines revealed low IC_50_ values (<30 μg·mL^−1^) for the CC-loaded NGs, as well as low toxicity of their unloaded analogues, indicating release of the protein cargo to the desired area. The obtained cell viability data, along with the observed internalization of our NGs into different cell lines, demonstrate the viability of our concept for a redox-responsive NG-based protein delivery platform.

## Figures and Tables

**Table 1:** Analysis of the tunability of the hydrodynamic diameter of NGs by varying the precursor ratios.

Entry	M_1_:M_2_	Size d (nm) ^1^	PDI ^1^	ζ-Potential (mV) ^1^
1	1:2.00	257 ± 1	0.11 ± 0.03	2.0 ± 0.7
2	1:1.50	263 ± 5	0.10 ± 0.03	3.0 ± 0.6
3	1:1.00	236 ± 7	0.08 ± 0.01	2.4 ± 0.4
4	1:0.50	185 ± 4	0.07 ± 0.03	2.3 ± 0.4
5	1:0.25	171 ± 8	0.03 ± 0.04	2.5 ± 0.1

^1^ Measured with a Zetasizer Nano-ZS at 25 °C in 10 mM PB.

**Table 2:** Analysis of cytochrome C-loaded NGs.

Entry	Theory wt.%	Determined wt.% ^1^	PLE (%) ^1^	Size d (nm) ^2^	PDI ^2^	ζ-Potential (mV) ^2^
1	5	4.3	85	242 ± 1	0.2 ± 0.01	−0.47 ± 0.01
2	10	8.7	87	406 ± 9	0.2 ± 0.01	−0.21 ± 0.06
3	20	7.5	38	450 ± 20	0.4 ± 0.01	−0.30 ± 0.08

^1^ Determined by UV absorption of cytochrome C at 410 nm; ^2^ measured with Zetasizer Nano-ZS at 25 °C in 10 mM PB.

**Table 3:** Calculated IC_50_ values for CC@NGs.

Cell Line	IC_50_ (μg·mL^−1^)
McF7	30.20
A549	29.93
HeLa	29.79

## Data Availability

Data are contained within the article or Supplementary Materials.

## Supplementary Materials

The following are available online at https://www.mdpi.com/article/10.3390/pharmaceutics13081276/s1: Figure S1. ^1^H-NMR (700 MHz, D_2_O) of NG; Figure S2. Full cryo-TEM of free NG; Figure S3. Change in size distribution of non-cleavable NGs after incubation at 37 °C in 10 mM PB with and without addition of 10 mM GSH at pH 7.4 measured by DLS; Figure S4. Full cryo-TEM of CC-loaded NG; Figure S5. CLSM of A549 cells; Figure S6. CLSM of HeLa cells; Figure S7. GPC analysis of dPG (**1**); Figure S8. ^1^H-NMR (700 MHz, MeOD) of 4-(6-methyl-1,2,4,5-tetrazin-3-yl) benzoic acid (**4**); Figure S9. ^1^H-NMR (700 MHz, MeOD) of dPG-metTet (**M_1_**); Figure S10. ^13^C-NMR (176 MHz, MeOD) of dPG-metTet (**M_1_**); Figure S11. IR of dPG–metTet (**M_1_**); Figure S12. ^1^H-NMR (400 MHz, MeOD) of bicyclo[2.2.1]hept-5-en-2-ylmethyl (4-nitrophenyl) carbonate (**6**); Figure S13. ^1^H-NMR (700 MHz, MeOD) of Norb–Cys-Norb (**M_2_**); Figure S14. ^13^C-NMR (176 MHz, MeOD) of Norb-Cys-Norb (**M_2_**); Figure S15. HRMS of Norb-Cys-Norb (**M_2_**) using electron spray ionization (ESI); Figure S16. UV/Vis spectrum of FITC–dPG–metTet.
