# Supplementary material for: Tunable Polyglycerol-Based Redox-Responsive Nanogels for Efficient Cytochrome C Delivery
Source: Pharmaceutics. 2021 Aug 17;13(8):1276. doi: 10.3390/pharmaceutics13081276 (PMC8397965; doi:10.3390/pharmaceutics13081276)
Supplement: Supplementary file 1 [file pharmaceutics-13-01276-s001.zip › pharmaceutics-1325623-supplementary.pdf]

pharmaceutics

Supplementary Information

Tunable Polyglycerol-Based Redox-Responsive Nanogels for Efficient Cytochrome C Delivery

Sebastian Schötz, Felix Reisbeck, Ann-Cathrin Schmitt, Mathias Dimde, Elisa Quaas, Katharina Achazi and Rainer Haag,*

Institute of Chemistry and Biochemistry, Freie Universität Berlin, Takustr. 3, 14195 Berlin, Germany; bastischoetz@zedat.fu-berlin.de (S.S.); freisbeck@zedat.fu-berlin.de (F.R.); schmia92@zedat.fu-berlin.de (A.-C.S.); matze111@zedat.fu-berlin.de (M.D.); equaas@zedat.fu-berlin.de (E.Q.); kachazi@zedat.fu-berlin.de (K.A.)

* Correspondence: haag@chemie.fu-berlin.de; Tel.: +49-30-838-52633

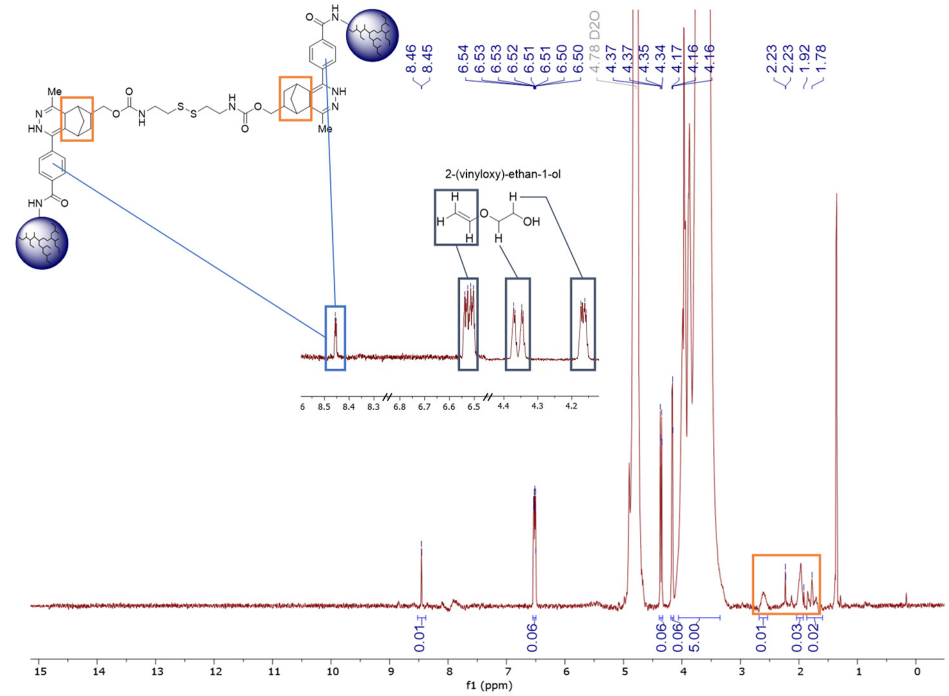

Figure S1: ^{1}H-NMR (700 MHz, D_2O) of NG. For this, the NGs (20 mg) were transferred into deuterated water. Norbornane signals are depicted in orange whereas the aromatic signals are depicted in light blue (inset). Signals referring to the protons of the quenching reagent are depicted in dark blue.

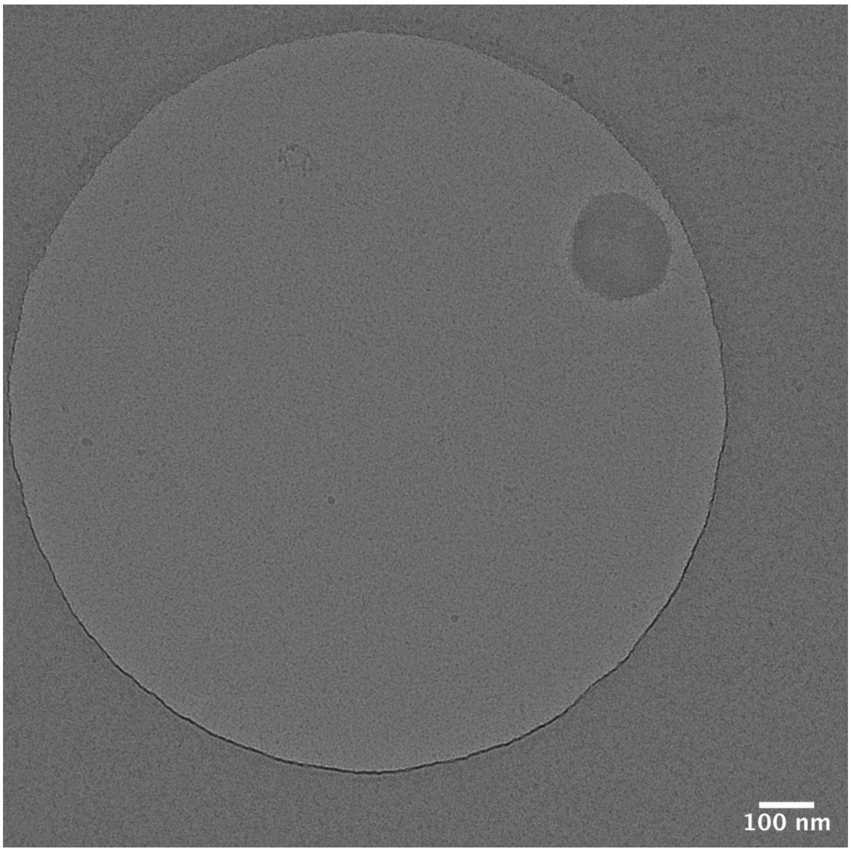

Figure S2: Full cryo–TEM of free NG.

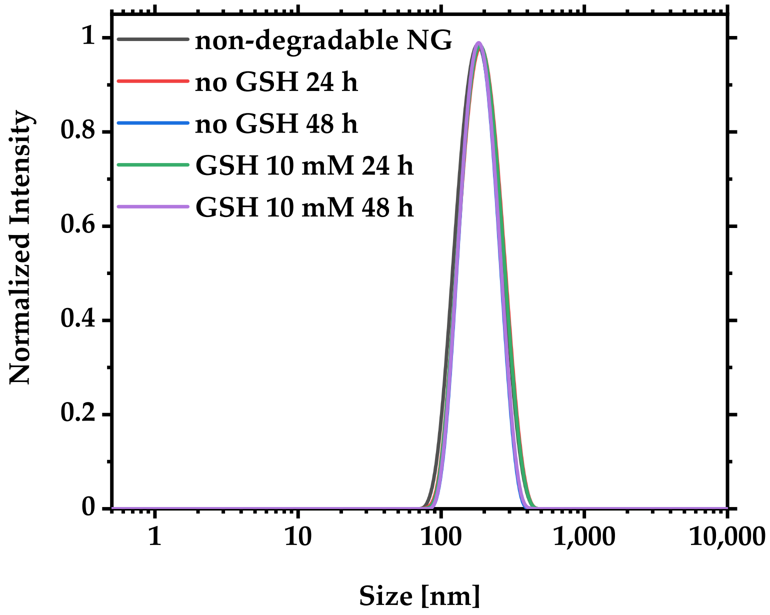

Figure S3: Change of Size distribution of non-cleavable NGs after incubation at 37 °C in 10 mM PB with and without addition of 10 mM GSH at pH 7.4 measured by DLS.

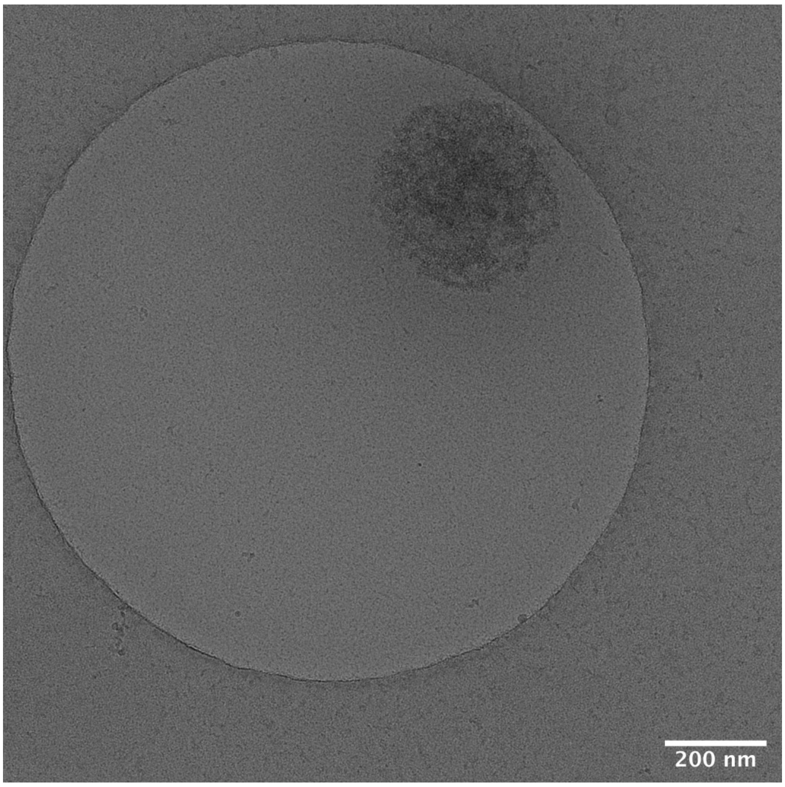

Figure S4: Full *cryo*–TEM of CC-loaded NG.

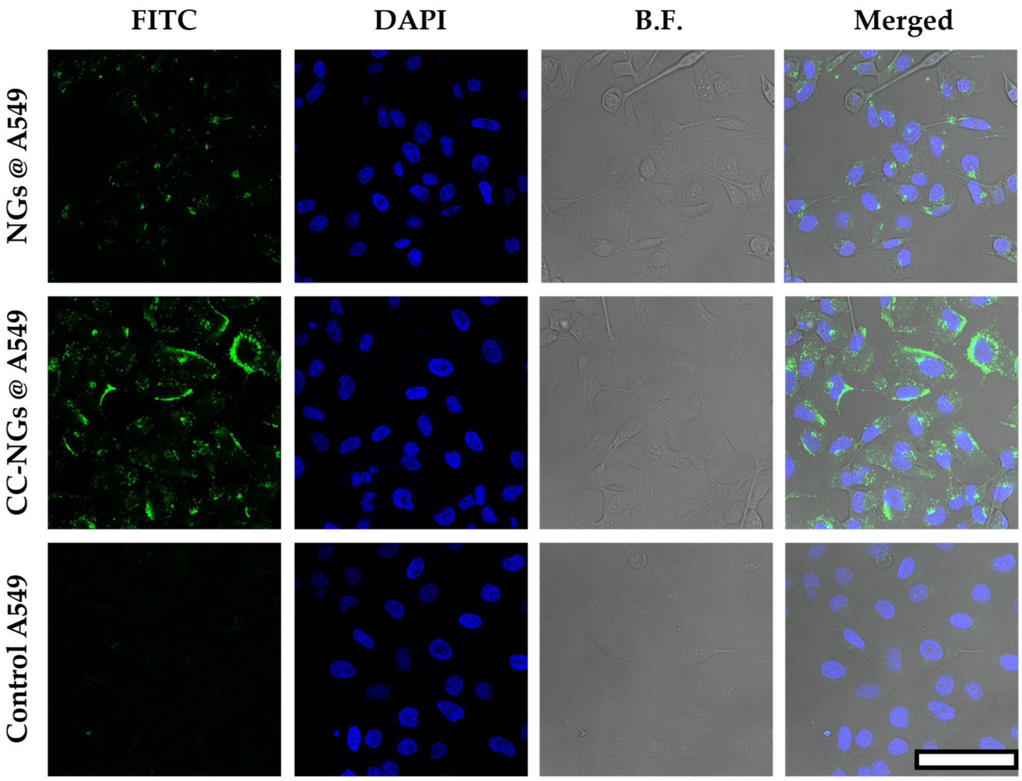

Figure S5: CLSM of A549 cells. Cells were incubated for 16 h at 37 °C with 0.5 mg mL^{-1} of loaded and unloaded FITC-labeled NGs. Non-treated cells were measured as a control (scale bar 75 µm).

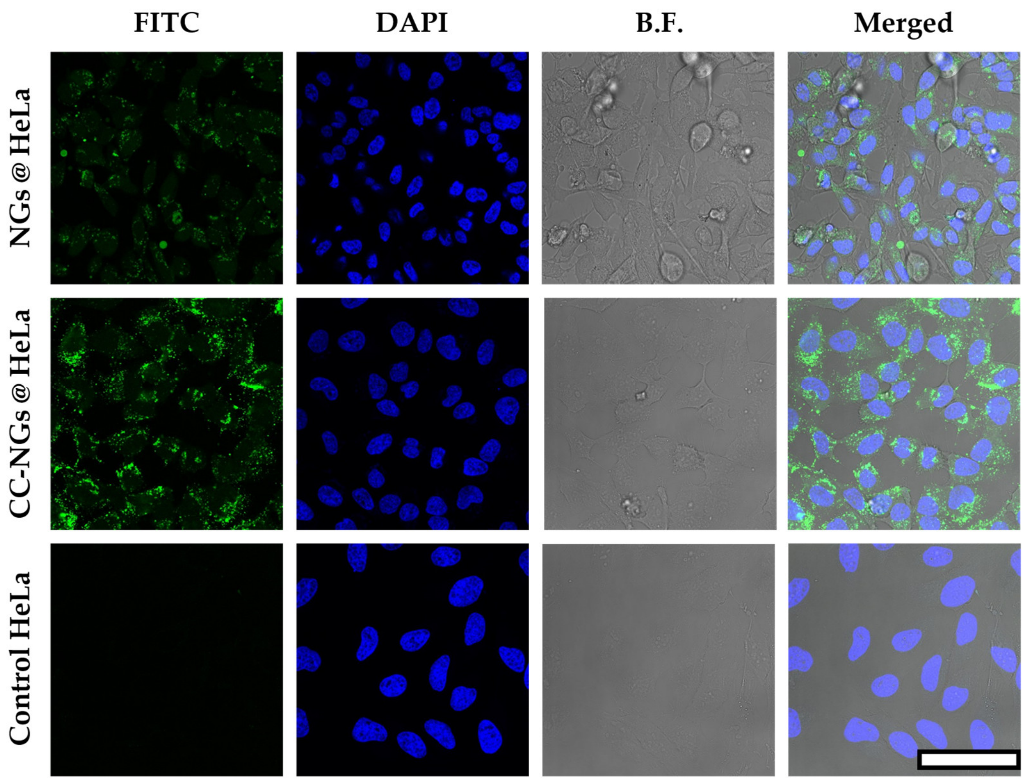

Figure S6: CLSM of HeLa cells. Cells were incubated for 16 h at 37 °C with 0.5 mg mL^{-1} of loaded and unloaded FITC-labeled NGs. Non-treated cells were measured as a control (scale bar 75 μm).

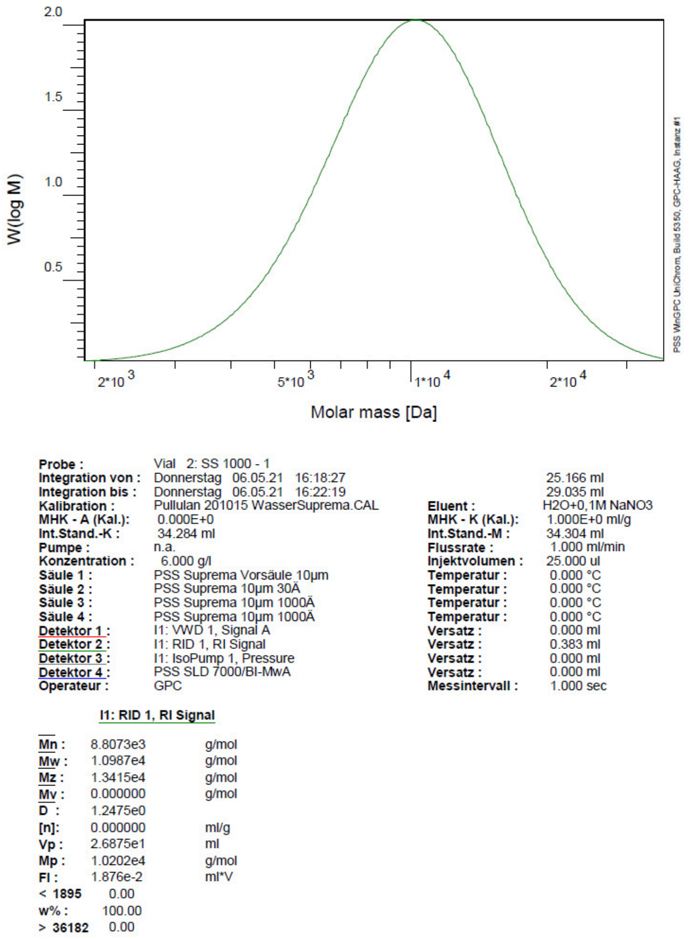

Figure S7: GPC analysis of dPG (**1**).

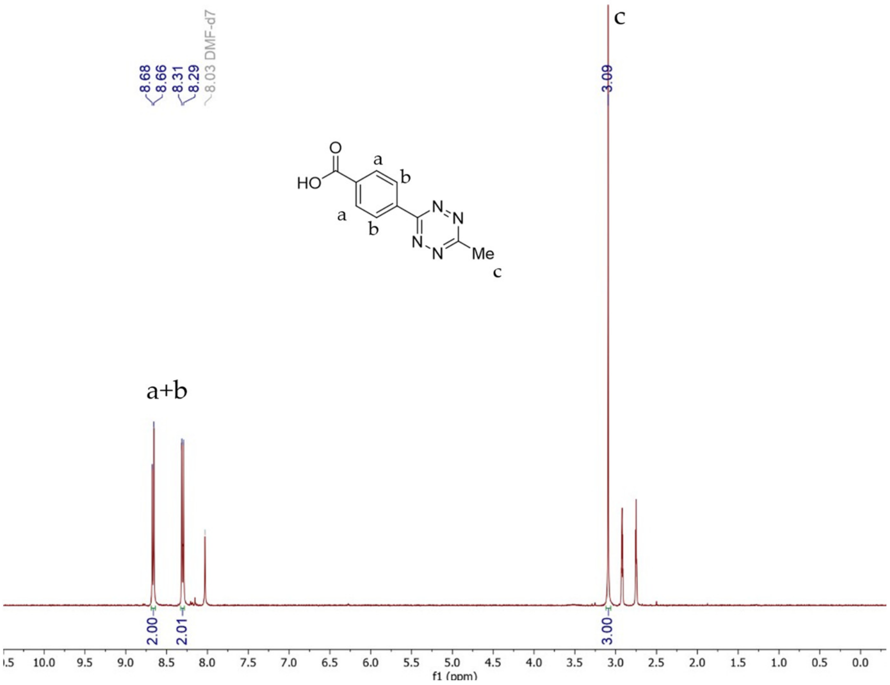

Figure S8: ^{1}H-NMR (700 MHz, MeOD) of 4-(6-methyl-1,2,4,5-tetrazin-3-yl) benzoic acid (**4**).

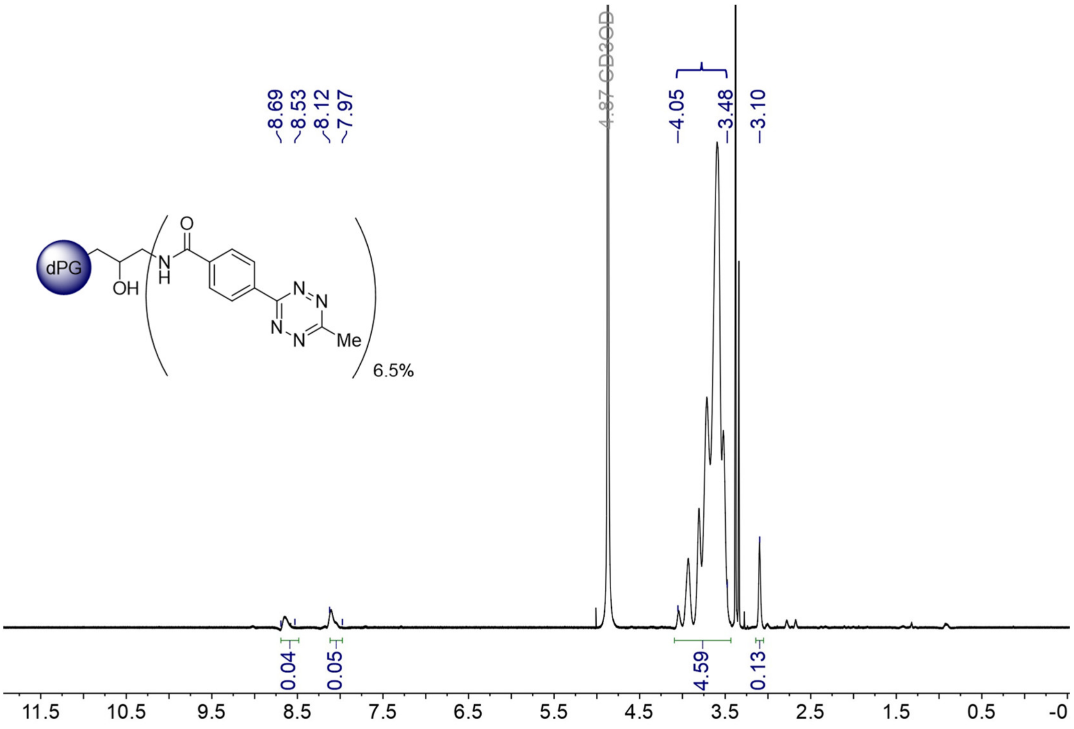

Figure S9: ^{1}H-NMR (700 MHz, MeOD) of dPG–metTet (**M$_1$**).

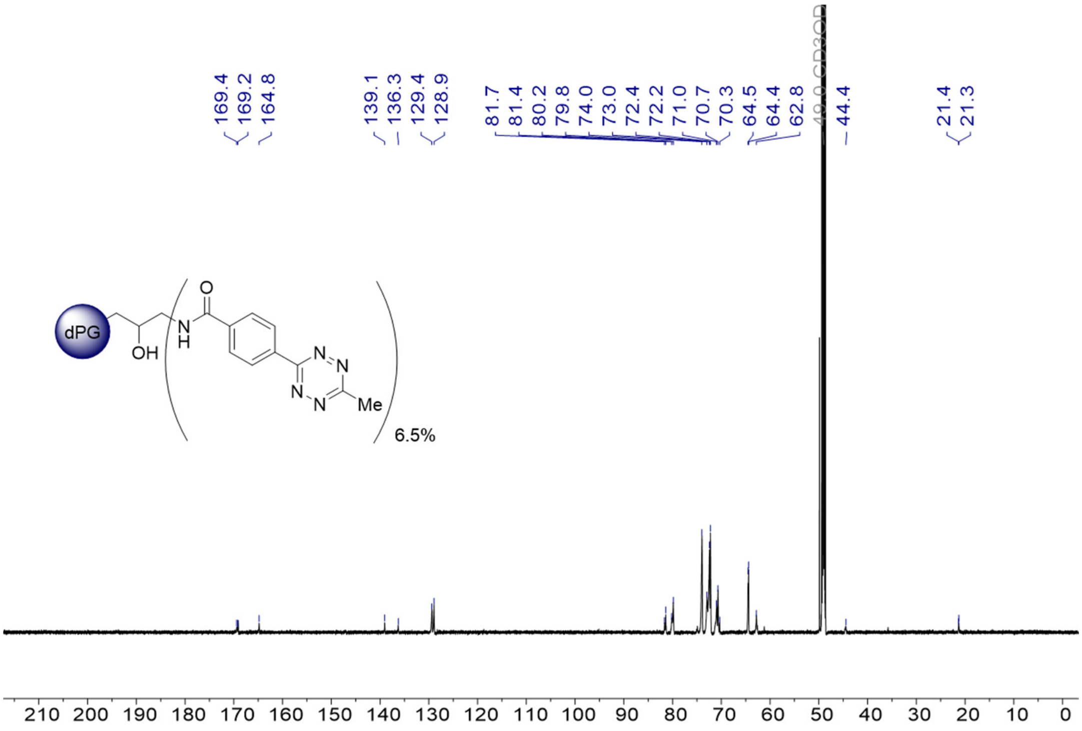

Figure S10: ^{13}C-NMR (176 MHz, MeOD) of dPG–metTet (**M$_1$**).

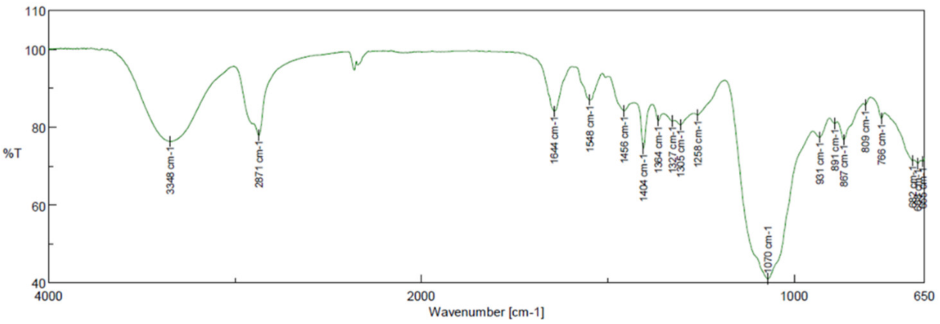

Figure S11: IR of dPG–metTet ($\mathbf{M_1}$).

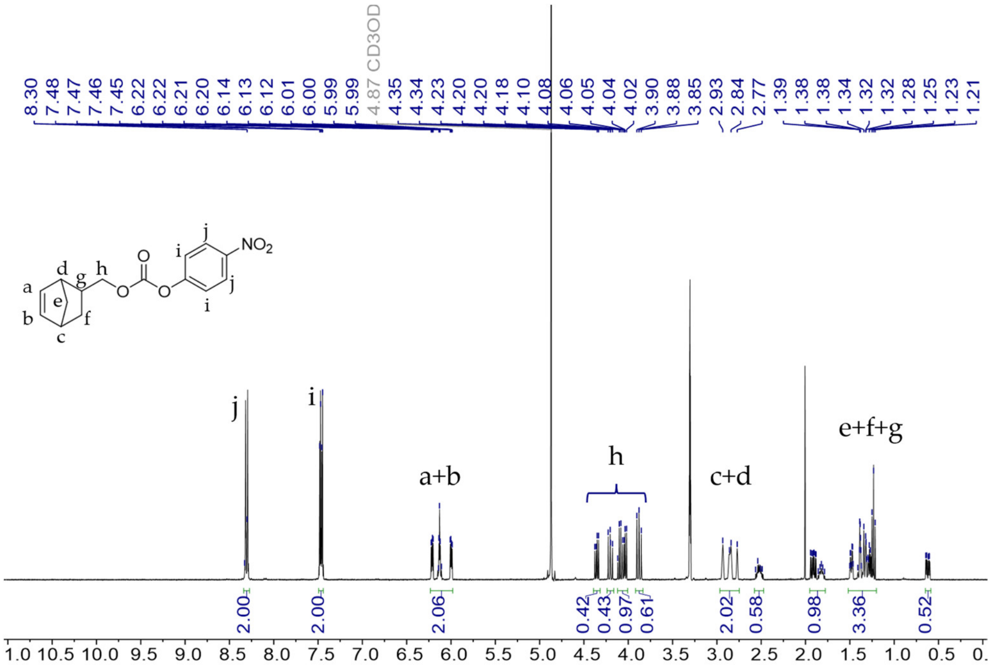

Figure S12: ^{1}H-NMR (400 MHz, MeOD) of bicyclo[2.2.1]hept-5-en-2-ylmethyl (4-nitrophenyl) carbonate (**6**).

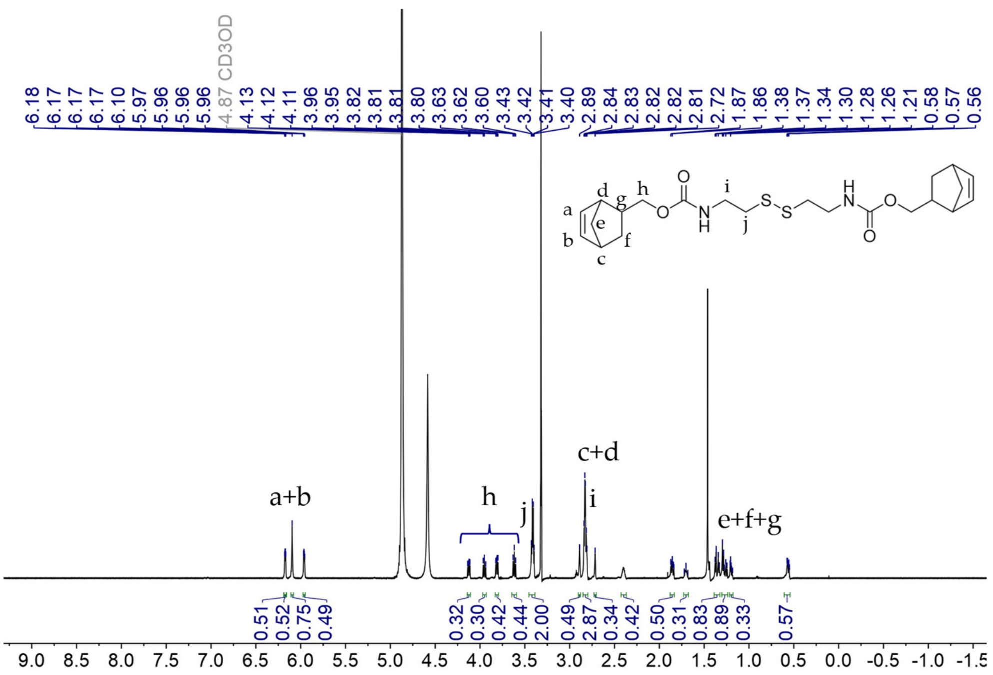

Figure S13: ^{1}H-NMR (700 MHz, MeOD) of Norb–Cys–Norb (**M**2).

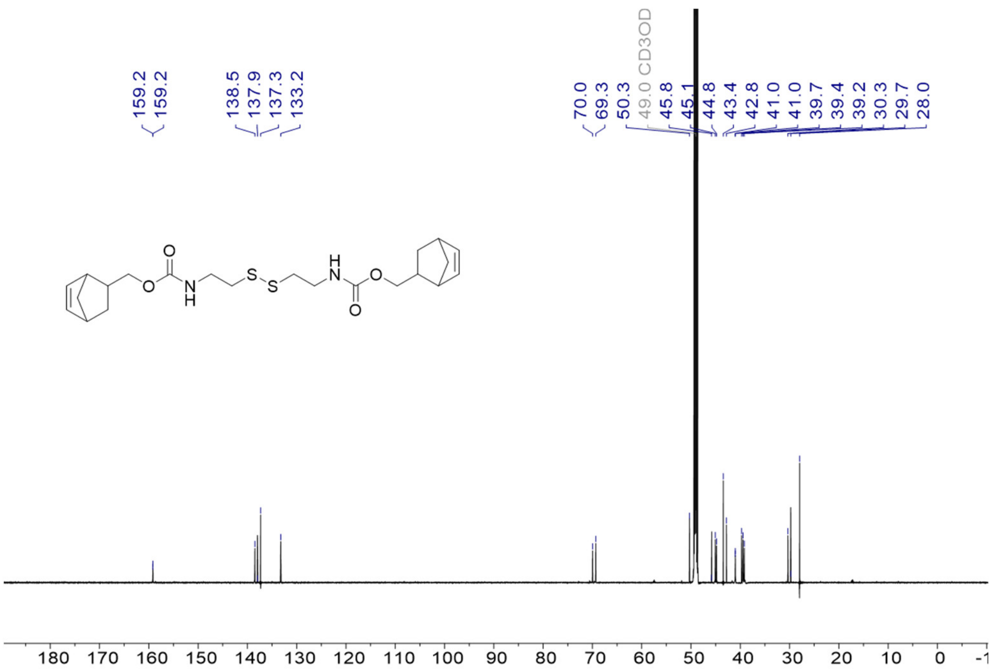

Figure 14: ^{13}C-NMR (176 MHz, MeOD) of Norb–Cys–Norb (**M**2).

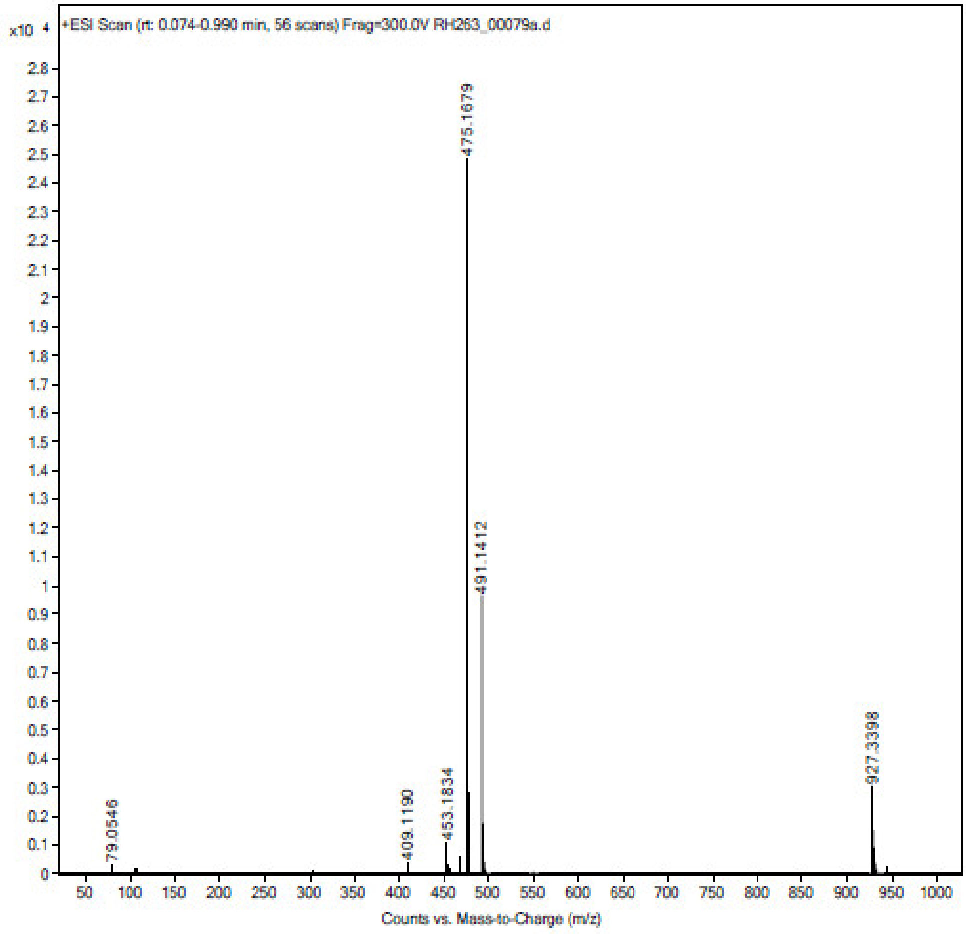

Figure S15: HRMS of Norb–Cys–Norb (**M**$_2$) using electron spray ionization (ESI).

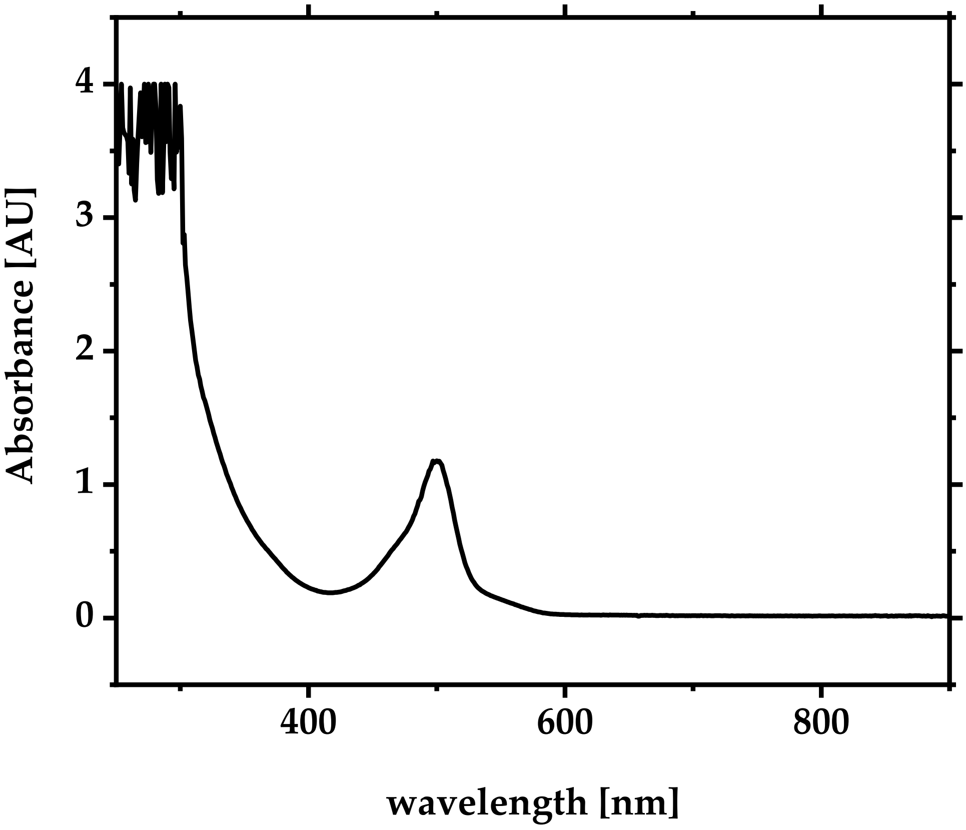

Figure S16: UV/Vis-Spectrum of FITC–dPG–metTet.
